# Arginase 2 and Polyamines in Human Pancreatic Beta Cells: Possible Role in the Pathogenesis of Type 2 Diabetes

**DOI:** 10.3390/ijms222212099

**Published:** 2021-11-09

**Authors:** Lorella Marselli, Emanuele Bosi, Carmela De Luca, Silvia Del Guerra, Marta Tesi, Mara Suleiman, Piero Marchetti

**Affiliations:** Department of Clinical and Experimental Medicine, University of Pisa, 56126 Pisa, Italy; bosiemanuele@gmail.com (E.B.); carmela.deluca3288@gmail.com (C.D.L.); s.delguerra@ao-pisa.toscana.it (S.D.G.); marta.tesi91@gmail.com (M.T.); mara.suleiman@for.unipi.it (M.S.); piero.marchetti@med.unipi.it (P.M.)

**Keywords:** arginase 2, polyamines, pancreatic islets, beta cells, type 2 diabetes

## Abstract

Arginase 2 (ARG2) is a manganese metalloenzyme involved in several tissue specific processes, from physiology to pathophysiology. It is variably expressed in extra-hepatic tissues and is located in the mitochondria. In human pancreatic beta cells, ARG2 is downregulated in type 2 diabetes. The enzyme regulates the synthesis of polyamines, that are involved in pancreas development and regulation of beta cell function. Here, we discuss several features of ARG2 and polyamines, which can be relevant to the pathophysiology of type 2 diabetes.

## 1. Introduction

Arginase is a manganese metalloenzyme that catalyzes the hydrolysis of L-arginine to L-ornithine and urea. There are two arginase isozymes in mammalians, ARG1 and ARG2 [1], that differ in tissue distribution, subcellular localization, immunological cross-reactivity and physiological function [2]. ARG1 is mainly expressed in hepatocytes, where it participates in the urea cycle, and is located in the cytosol [3]. ARG2 is expressed at different levels in extra-hepatic tissues, and is located in the mitochondria [4], where it converts L-arginine to L-ornithine [4]. The enzyme plays a role in nitric oxide and polyamine metabolism, which are involved in a variety of distinct physiological processes [5]. In recent years, there has been a surge of publications linking arginase to various aspects of health, including metabolic processes [5] and the immune system [6,7,8]. Accordingly, investigations of this enzyme have offered new perspectives on ad hoc designed therapeutic strategies [9,10,11]. In this review, we focus on the presence and possible role of ARG2 and polyamines in human pancreatic beta cells, which may be relevant to the pathophysiology of type 2 diabetes (T2D).

## 2. ARG2 Gene

### 2.1. Locus, Expression and Tissue Specificity

ARG2 gene maps to chromosome 14q24.1 (chr14:67619920-67651708; GRCh38.p13), spans 31,789 nt in the plus strand. Alternative splicing of the RNA generates four transcript variants, while only one (ARG2-201, NM_001172.4) encodes the protein (P78540). The protein coding transcript (ENST00000261783.4) includes 8 exons, it is 1911 nt in length (5′ UTR region: 58 nt, coding sequence: 1065 nt, 3′ UTR region: 788 nt). The encoded peptide has a sequence of 354 amino acids (NP_001163.1), shares 59% sequence identity with ARG1, and differs from this latter for the presence of a transit peptide (aminoacids 1–22) that is necessary for the translocation of the enzyme inside the mitochondria.

**Figure 1:**
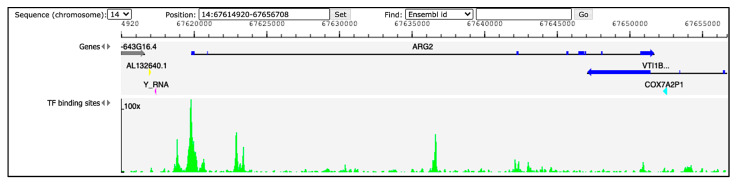
Peaks of TF binding sites from GTRD (http://gtrd.biouml.org, v20.06, accessed on 31 August 2021). The tracks in the diagram indicate genes and TF binding sites. Dark-blue segments in the ARG2 gene correspond to the exons. The green histograms refer to DNA footprint peaks corresponding to the TF binding sites in the region chr14:67,614,920–67,656,708.

Hints regarding ARG2 regulation in humans can be derived from available ChIP-seq and DNase-seq experiments. The Gene Transcription Regulation Database (GTRD; http://gtrd.biouml.org, accessed on 31 August 2021) reports data of transcription factor (TF) binding sites derived from these procedures [12]. Information for ARG2 region ± 5000 bp retrieved from GTRD v20.06 identifies multiple peaks (corresponding to TF binding sites) upstream the gene (Figure 1). Among them, the three with the highest signal intensity correspond to the binding site of the transcription factors HMG-box containing 4 (HMGXB4), HMG-box transcription factor 1 (HBP1) and zinc finger protein 35 (ZNF35), possibly involved in the regulation of ARG2. Validated regulators would be crucial to obtain insights into the role of ARG2 in different cell types.

**Figure 2:**
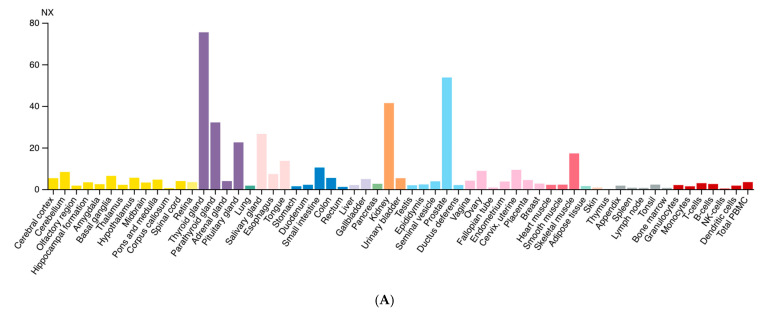

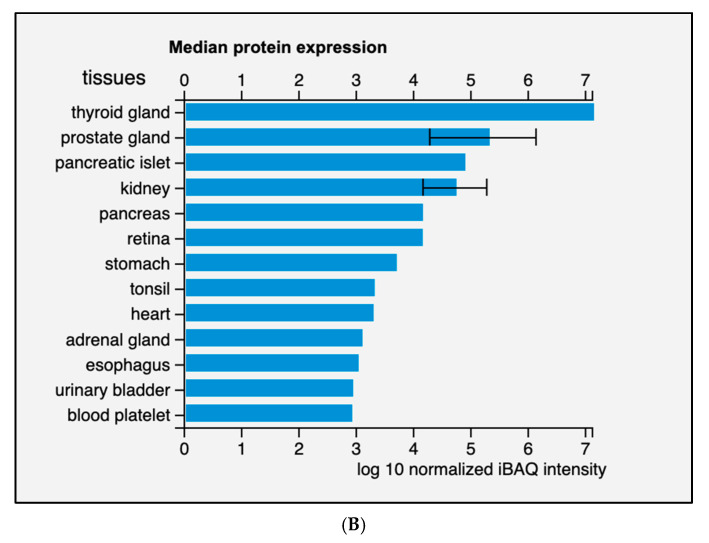
Expression of ARG2 in human tissues. (**A**) Histogram of the Consensus Normalized eXpression (NX) of ARG2 transcript in human tissues as reported in the Human Protein Atlas v20.1 (https://www.proteinatlas.org/ENSG00000081181-ARG2/tissue; accessed on 31 August 2021). Color-coding is based on tissue groups, each consisting of tissues with functional features in common. (**B**) Histogram of ARG2 protein expression as reported in Proteomics DB v3.0 (https://www.proteomicsdb.org/; accessed on 31 August 2021) [16,17]. Organ and tissues with the higher protein expression are thyroid and prostate glands, pancreatic islets, kidney, and pancreas. iBAQ: intensity Based Absolute Quantification.

Diversely from ARG1, which is primarily expressed in the liver, ARG2 expression is much more variegated [13]. Morris et al. detected ARG2 transcripts in almost every tissue tested from human and mouse [14], while the presence of multiple isoforms was detected in human samples. Data from the Human Protein Atlas v20.1 [15] confirm that ARG2 transcript is almost ubiquitously expressed (Figure 2A); tissues with higher levels of ARG2 transcript and protein are endocrine glands, prostate, and kidney (Figure 2B).

**Figure 3:**
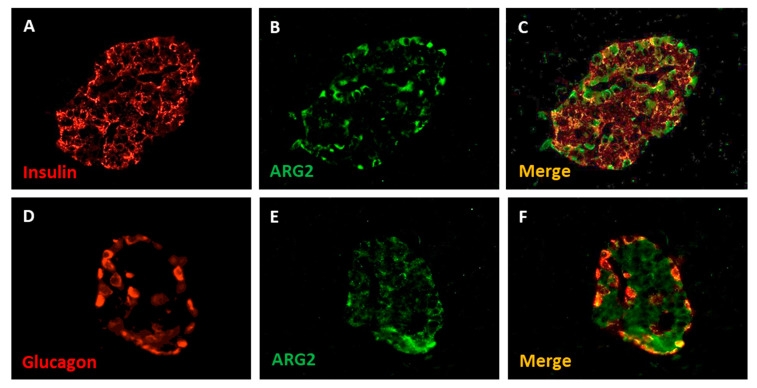
Immunofluorescence detection of insulin (**A**), glucagon (**D**) and ARG2 (**B**,**E**) in human pancreatic islet cells. The enzyme is localized in insulin-containing beta cells (**C**) and, to a lower extent, in glucagon-contaning alpha cells (**F**). Magnification 40×. Adapted from Solimena et al., 2018 [18].

In pancreatic tissue, ARG2 protein was detected in a subset of insulin-positive and glucagon-positive cells by immunofluorescence staining, and it was rare in acinar cells (Figure 3) [18].

### 2.2. Genetic Variations of ARG2 and Associated Traits

**Figure 4:**
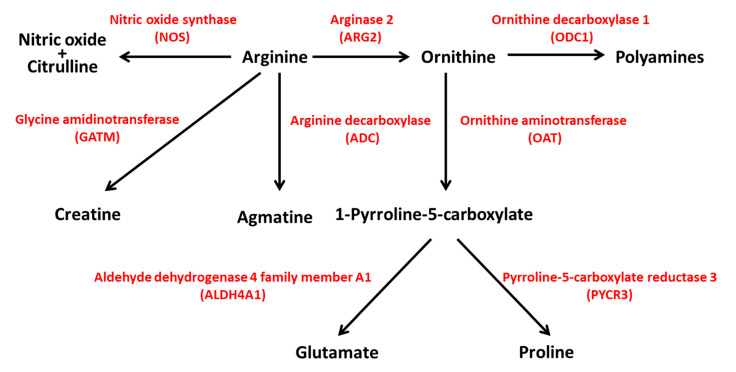
ARG2 activity is central to different pathways in human metabolism. ARG2 is involved in the biosynthesis of polyamines, glutamate, proline, creatine, agmatine, nitric oxide and citrulline. NOS, nitric oxide synthase; ARG2, arginase 2; ODC1, ornithine decarboxylase 1; GATM, glycine amidinotransferase; ADC, arginine decarboxylase; OAT, ornithine aminotransferase; ALDH4A1, aldehyde dehydrogenase 4 family member A1; PYCR3, pyrroline-5-carboxylate reductase 3.

The Genome Aggregation Database (GnomAD v.2.1.1; https://gnomad.broadinstitute.org/; accessed on 31 August 2021) reports 592 genetic variants of ARG2 in ~141 k samples from whole-exome and genome sequencing studies [19]. Most of the variants are predicted to have limited effects on gene function (Supplementary Table S1). Protein-truncating variants such as stop gained, frameshift and splice acceptor/donor are few and have low (≤10^−5^) allele frequency (MAF). More common variants (MAF ≥ 10^−4^) are likely neutral, as they fall in intronic or untranslated regions, or have limited effects on ARG2 coding sequence (missense, synonym and inframe deletion variants). The analysis of the GnomAD ‘control’ subset, including ~60 k samples from individuals selected as controls in case/control studies of common disease, identified 352 variants, with 20 being predicted as loss-of-function (LoF). In both the GnomAD datasets, there are no homozygous individuals for LoF alleles. In humans, an ARG2 variant has been reported as pathogenic (or likely pathogenic); it regards one allele (rs742869) that has been linked to an increased risk for Alzheimer’s disease [20]. However, the fact that the corresponding allele frequency (0.46) is the highest among all the variants reported for ARG2 in GnomAD suggests that such an association might be a false positive. In ARG2-KO mice, hyperargininemia in homozygous or heterozygous mutants has been observed, together with diminished fertility in males [21]. The genetic interaction between ARG1 and ARG2 has been studied in mice with individual or combined homozygous inactivation of these genes [22]. ARG1-KO mice displayed severe hyperammonemia and poor prognosis, and ARG2-KO mice exhibited hyperargininemia [22], consistent with previous data [21]. Double knockout mice showed a shorter life span than ARG1-KO mice, suggesting a key, although still poorly understood, role of ARG2 in the modulation of life expectancy [22]. Considering the centrality of arginine for the biosynthesis of polyamines, glutamate, proline, agmatine, creatine, nitric oxide (NO) and citrulline (Figure 4), the hydrolysis of arginine by ARG2 is likely to exert a plethora of effects through different pathways with implications for distinct physiologic processes. In the next paragraph, we will describe how ARG2 in islet beta cells could be implicated in the pathogenesis of type 2 diabetes.

## 3. ARG2 and the Pancreatic Beta Cells

ARG2 is expressed in human islets and beta cells, and it is downregulated in T2D, as indicated by transcriptome studies. ARG2 expression was found to be reduced by more than 1.5-fold in islets of T2D subjects as compared to non-diabetic controls in three independent organ donor cohorts [18,23,24] and one cohort of pancreatectomized patients [18], achieved by microarray [18,23] or RNA-seq [24] analysis. ARG2 gene quantification, assessed in isolated beta and alpha cell-enriched fractions obtained from five non-diabetic and four T2D organ donors, showed the enrichment of ARG2 in beta cells compared to alpha cells, and the downregulation of the gene in beta cells of T2D subjects [18].

Detailed information on the expression of ARG2 transcript in the human pancreatic cell subtypes is provided by RNA-seq analysis. Data on the expression of individual cells permit us to calculate the percentage of the cell subtype expressing the gene [25]. ARG2 mRNA was detected in 66% of beta cells, 38% of alpha cells, 55% of PP cells, and 37% of duct cells. The proportion of acinar cells expressing ARG2 was 9% [25]. In the same study, ARG2 mean RPKM (Reads Per Kilobase per Million mapped reads) values were 66.06 in beta cells, 24.33 in alpha cells, 47.61 in PP cells, 20.25 in duct cells, and 6.28 in acinar cells; no signal was detected in delta cells. These data recognize ARG2 as a gene preferentially expressed in beta cells. A significantly higher expression of ARG2 in human beta cells has been demonstrated in additional studies [26,27,28].

The expression of ARG2 was also investigated in pancreas single cells obtained from T2D subjects, showing no difference in beta cells of T2D subjects [27,29]. In addition, a more recent study did not detect any significant difference in ARG2 signal between beta (avelogCPM: 5.64) and alpha (avelogCPM: 5.53) cells of T2D subjects [28].

The association between ARG2 expression and human beta cell function was investigated by correlation analyses in a few studies. A positive association between ARG2 expression and the insulin stimulation index values was found in islets of 58 non-diabetic and 19 T2D organ donors [18]. This positive association has been also observed in other studies [30]. Accordingly, HbA1c levels of organ donors were reported to be negatively correlated to islet ARG2 expression [30,31]. 

In summary, ARG2 is predominantly expressed in human beta cells, and its expression seems to favor beta cell function.

## 4. Polyamines and the Pancreatic Beta Cell

### 4.1. Polyamine Synthesis and Catabolism

One of the roles of ARG2 is the regulation of polyamine biosynthesis. Polyamines are ubiquitous, polycationic aliphatic amines involved in many physiological functions. They play an important role in cell growth, proliferation, and differentiation. [32,33,34]. In cells at physiological pH, they are fully protonated, and are therefore able to bind to negatively charged molecules, such as DNA, RNA, specific types of proteins and phosholipids [35], thus playing a role in DNA stability, regulation of gene transcription and mRNA translation, protein and nucleic acid synthesis [33,36]. Other polyamine functions include the regulation of several ion channel activities and the modulation of cell membrane receptors with effects on specific signaling processes [33,36]. In addition, possible sites of physiological polyamine actions are the cell–cell interactions mediated via cadherins or Toll-like receptors, as well as the cytoskeleton and the microtubule networks [36].

**Figure 5:**
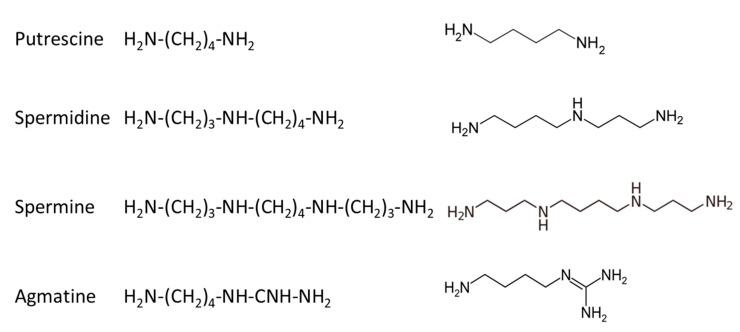
Structure of polyamines identified in mammalian cells.

In mammalians, there are three basic polyamines: putrescine, spermidine and spermine, while a fourth compound, agmatine, has been more recently identified and studied [32,33,34]; their structure is given in Figure 5.

**Figure 6:**
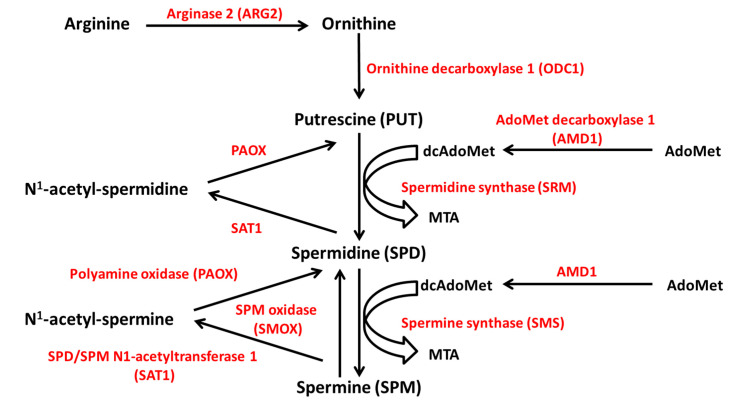
Polyamine metabolism. Polyamines are synthesized from ornithine, which derives from arginine through the reaction of ARG2. Ornithine is converted to putrescine by ODC1. Spermidine and spermine derive from putrescine by successive attachment of two propylamine groups by the action of SRM and SMS, respectively. The propylamine group donor is the decarboxylated S-adenosylmethionine (dcAdoMet), derived from S-adenosyl-methionine (AdoMet) by the action of AMD1. Polyamine catabolism depends on the acetylation of spermine and spermidine by SAT1, and the removal of the aminopropyl moiety by PAOX. Spermine can be oxidated to spermidine by SMOX. ODC1, ornithine decarboxylase 1; SRM, spermidine synthase; SMS, spermine synthase; AMD1, AdoMet decarboxylase 1; SAT1, SPD/SPM N1-acetyltransferase 1; PAOX, polyamine oxidase; SMOX, SPM oxidase. MTA, 5′-methylthioadenosine.

The source of polyamines may be exogenous, through active dietary intestine uptake, or endogenous, through intracellular de novo synthesis and interconversion pathways [34]. The polyamines that are found in the gut lumen are mainly provided by food and the microbiome. They undergo intensive metabolization before entering the systemic circulation, so that only a small proportion reaches the plasma [37]. Endogenously, polyamines are synthesized from ornithine, which derives from the amino acid arginine through the reaction of ARG2 (Figure 6). The subsequent reaction catalyzed by the enzyme ornithine decarboxylase 1 (ODC1), the limiting enzyme in polyamine synthesis, produces putrescine. Spermidine and spermine derive from putrescine by successive attachment of two propylamine groups by the action of aminopropyl-transferases, namely spermidine synthase (SRM) and spermine synthase (SMS). The propylamine group donor is the decarboxylated S-adenosylmethionine (dcAdoMet), derived from S-adenosyl-methionine (AdoMet) by the action of adenosylmethionine decarboxylase 1 (AMD1), another limiting enzyme [36].

Polyamine catabolism largely depends on the acetylation of spermine and spermidine by spermine/spermidine N1-acetyltransferase 1 (SAT1), an inducible, limiting enzyme for polyamine conversion. SAT1 transfers the acetyl group from acetyl-coenzyme A (acetyl CoA) to the N1 position of spermine and spermidine with production of N1-acetylspermine and N1-acetylspermidine. The aminopropyl moiety is removed by the enzyme polyamine oxidase (PAOX) and the acetylated substrates are converted into spermidine and putrescine, respectively. Furthermore, spermine can be oxidized to spermidine by the enzyme spermine oxidase (SMOX) (Figure 6) [38,39]. In addition to the conversion pathway, a catabolic branch of polyamine metabolism exists, in which the enzyme diamine oxidase (DAO) catalyzes the polyamine oxidative deamination [38].

Therefore, polyamine metabolism is tightly regulated by the activity of ODC1, AMD1 and SAT1, three rapid-turnover enzymes controlled at different levels. ODC1 activity is increased in response to growth factors, amino acids, or hypotonic stress, and is tightly regulated at the level of gene transcription, mRNA translation and degradation, and protein catabolism [40]. In turn, polyamines regulate the efficiency of ODC1 mRNA translation by affecting the rates of initiation and elongation. A post-translation mechanism of control involves the induction of a regulating protein, the ornithine decarboxylase antizyme (OAZ). OAZ is induced by the intracellular accumulation of polyamines, which regulate OAZ mRNA translation. OAZ forms a complex with ODC1, induces a conformational modification of the enzyme resulting in its inhibition and acceleration of its catabolism [41]. AMD1 is negatively modulated by polyamines, which act at mRNA transcription and translation levels and on AMD1 protein half-life [42]. Finally, SAT1 is induced by the increase in the intracellular polyamine content [43], favors the excretion of polyamines in the acetylated form, and enhances the catabolism of putrescine by DAO [33].

Other mechanisms may affect polyamine pathway activity. Ornithine concentration in normal tissue is lower than the *Km* of ODC1, suggesting that the enzyme functions efficiently below its *Vmax* value [33]. Therefore, any modification that influences the intracellular ornithine concentration (for example variation in the utilization of arginine to form ornithine or in the activity of enzymes of ornithine metabolism other than those involved in the polyamine synthesis) can affect the polyamine biosynthetic pathway [33]. In addition, in arginine-depleted endothelial cells, the induction of putrescine uptake is totally inhibited by arginine, but only partially by ornithine, suggesting that arginine plays a major regulating role [44].

### 4.2. Role of Polyamines in Beta Cells

Polyamines have been detected in mouse and rat pancreatic islets, mainly located in the secretory granules of beta cells [45,46], and have been implicated in proinsulin biosynthesis and insulin secretion [47].

In pancreatic islets, polyamines show a high spermine–spermidine ratio [46]. Chronic exposure (24 or 48 h) of rat islets to high glucose concentration (20 mM) stimulated the synthesis of putrescine and spermidine, rather than spermine [46]. Welsh N and coll. showed that the culture of mouse islets in 16.7 mM glucose concentration for 48 h prevented spermidine content decrease occurring after the islet isolation procedure and increased the synthesis and content of spermine [48,49]. Importantly, the same authors observed that depletion of putrescine, spermidine and spermine in isolated mouse islets was associated with reduced glucose-stimulated insulin release, insulin content, insulin transcription and DNA synthesis [48,49].

The mechanisms possibly involved in the modulation of beta cell function by polyamines have been explored in a few studies. Inhibition of spermidine synthesis induced a decrease in glucose-stimulated insulin secretion associated with inhibition of the rise of cytoplasmic Ca^2+^ concentration in mouse Beta-TC6 cells. These defects were partially reversed by adding spermidine [50]. Conversely, spermine decreased the free-Ca^2+^ concentration in the incubation medium of pancreatic islets of ob/ob mice, likely through a stimulatory effect on Ca^2+^ uptake in the mitochondrial fraction of beta cells [51]; the effects of a pro-inflammatory stimulus on the content of polyamines in islets or beta cell lines were also investigated. The exposure of rat islets or RINm5F cells to interleukin 1 beta determined a decrease in the cellular content of spermidine and spermine and a reduction in the cell replication activity [52]. Conversely, Smismans and coll. have reported that interleukin 1 beta caused increased ODC activity in rat insulinoma (RIN) cells associated with an increased cellular content of the enzymatic product putrescine [53].

In vivo studies in animal models confirmed what observed in in vitro settings. Pancreas and islet depletion of spermidine and spermine were accompanied by decreased insulin production and impaired glucose-stimulated insulin secretion in transgenic mice overexpressing Sat1, the enzyme regulating the polyamine catabolism [54].

In addition, inhibition of deoxyhypusine synthase (DHPS), which mimics the effect of spermidine depletion on the hypusine modification of the eukaryotic initiation factor 5A (eIF5A), was associated with reduced beta cell mass, partial loss of beta cell function, and development of diabetes in high-fat diet fed mice [55].

In the same line, exogenous administration of L-arginine or spermidine in rats with alloxan-induced diabetes resulted in an improvement of glycaemia and a reduction of glycosylated hemoglobin (HbA1c) levels [56]. In this animal model, the administration of arginine, putrescine, spermidine or spermine was associated with beta cell protection [57]. Interestingly, it has been recently reported an association between the serum levels of polyamines and T2D in a cohort of patients [58]. In particular, it was found that serum putrescine levels were significantly elevated in patients with T2D compared to non-diabetic subjects and correlated with HbA1c levels. Instead, a positive association was found between fasting insulin levels and serum spermine [58].

Morphological, functional and molecular assessments with the use of human pancreatic samples, isolated islets and laser-capture microdissected beta cells were also performed to further investigate the role of polyamines in beta cell pathophysiology [59,60]. The results showed insulin-positive cells in islets of non-diabetic and T2D subjects and spermine detection in islet cells of non-diabetic subjects, but not in those of T2D donors. Acute stimulation of islets with the polyamine synthesis inhibitor DFMO determined a marked decrease of glucose-stimulated insulin secretion. The quantitative expression of beta cell ARG2, ODC1 and SAT1, assessed by real-time PCR, showed a significant downregulation of ARG2 and ODC1 in beta cells of T2D subjects compared to non-diabetic controls. No significant difference was observed in the expression of SAT1, although the mean of the relative expression was higher in beta cells of T2D subjects compared to that of non-diabetic controls. These results confirmed that the expression of ARG2, which synthesizes ornithine from arginine, is reduced in beta cells of T2D subjects, and showed that also ODC1, that promotes polyamine synthesis from ornithine, is downregulated in such cells. This may lead to reduced polyamine content in insulin secreting cells, hampering the insulin secretion process. Accordingly, DMFO, an inhibitor of ODC1, reduced glucose-stimulated insulin release.

## 5. Conclusions

This article reviews the available information on the molecular and functional properties of ARG2 in human pancreatic beta cells, where ARG2 is expressed at lower levels in the case of T2D. This, together with downregulation of ODC1, could lead to reduced polyamine synthesis which, in turn, causes impaired insulin secretion. These observations should foster further studies to understand whether ARG2 might be a target for the prevention of beta cell functional damage.

## Data Availability

Not applicable.

## Supplementary Materials

The following are available online at https://www.mdpi.com/article/10.3390/ijms222212099/s1.
